# Low expression of phosphatase and tensin homolog in clear-cell renal cell carcinoma contributes to chemoresistance through activating the Akt/HDM2 signaling pathway

**DOI:** 10.3892/mmr.2015.3740

**Published:** 2015-05-07

**Authors:** JUN CHEN, HE ZHU, YAN ZHANG, MAN-HUA CUI, LI-YING HAN, ZHAN-HUI JIA, LING WANG, HONG TENG, LI-NING MIAO

**Affiliations:** 1 Department of Gynaecology and Obstetrics, The Second Hospital of Jilin University, Changchun, Jilin 130041, P.R. China; 2 Department of Breast Surgery, The Second Hospital of Jilin University, Changchun, Jilin 130041, P.R. China; 3 Department of Nephrology, The Second Hospital of Jilin University, Changchun, Jilin 130041, P.R. China

**Keywords:** clear-cell renal cell carcinoma, chemoresistance, phosphatase and tensin homolog, Akt/HDM2 signaling, etoposide

## Abstract

Clear-cell renal cell carcinoma (CCRCC) is the most frequent primary malignancy in the adult kidney. Most patients with advanced CCRCC have poor prognosis as CCRCC remains resistant to chemotherapy. The present study explored the possible mechanism underlying CCRCC resistance to chemotherapy and found that loss of PTEN in CCRCC may be involved. Knockdown of PTEN in the CCRCC cell line ACHN blocked etoposide-induced apoptosis and etoposide-impaired cell proliferation was also inhibited. It has been demonstrated that most chemotherapy drugs exert their anti-cancer effects via p53-mediated apoptosis, and in accordance, with this, the present study showed that treatment with etoposide significantly increased p53 levels. Silencing of PTEN in ACHN inhibited the Akt/HDM2 signaling cascade and depressed p53 expression, and the interaction between HDM2 and p53 was also enhanced. This was further verified in CCRCC tissue specimens from patients The results of the present study suggested that loss of PTEN, which deactivated Akt/HDM2 signaling followed by degradation of p53, may contribute to the development of etoposide resistance in CCRCC.

## Introduction

Renal cell carcinoma (RCC) is the most frequent primary malignancy in the adult kidney, accounting for 3% of all adult tumors. Each year, >200,000 individuals are diagnosed with this type of cancer all over the world and >100,000 succumb to it (1,2). Clear-cell renal-cell carcinoma (CCRCC) is the most common type of RCC, which accounts for ~80% of all cases of RCC (3). CCRCC is highly aggressive and resistant to conventional chemotherapy (4). At present, the most efficient treatment for CCRCC is surgical resection. In the case of localized tumors, this therapy potentially cures affected patients. However, most patients have developed distant metastases at the time of diagnosis. Distant metastasis and local recurrence frequently occurs in 1/3 of patients receiving radical surgical treatment, among whom only 4–6% are sensitive to chemotherapy. Therefore, in spite of the significant progress made in recent years in improving surgical technology and chemotherapy, the five-year survival rate of CCRCC remains low. Thus, it is urgently required to elucidate the associated mechanism underlying chemoresistance in CCRCC and provide a basis for developing novel efficient approaches for CCRCC treatment.

Phosphatase and tensin homolog (PTEN) is a dual-specificity phosphatase with protein phosphatase and lipid phosphatase activity, and PTEN was the first phosphatase identified as a tumor suppressor. It was reported that PTEN participates in multiple signaling pathways and has important roles in regulating cell growth, apoptosis, adhesion, migration and invasion (5). Increasing evidence demonstrated that PTEN has a vital role in tumor development, and the absence or mutation of PTEN was frequently discovered in various tumors, including CCRCC (6–8). Mutation of the PTEN in mice leds to high susceptibility to cancer (9). Furthermore, tissue-specific deletion of PTEN in breast, skin and prostate resulted in tumorigenesis (10,11). PTEN mutations are rarely present in CCRCC; however, decreased PTEN protein expression levels were detected, suggesting that PTEN is involved in CCRCC development.

Akt is a member of the serine/threonine protein kinase family, which has an important role in regulating cell proliferation and apoptosis (12). It has been demonstrated that Akt is activated in numerous malignant tumors, including colorectal (13), ovarian (14), endometrial (15) and thyroid cancer (16) as well as CCRCC (17). Activated Akt inhibits cell apoptosis and promotes cell proliferation through phosphorylating downstream substrates, including B-cell lymphoma 2-associated death promoter (18), caspase-9 (19,20), nuclear factor-κB kinases (21,22) and HDM2 (23). PTEN is a specific antagonist of phosphatidylinositol(3,4,5)-trisphosphate (PIP3), which blocks phosphoinositide 3-kinase (PI3K)/Akt signaling through dephosphorylating PIP3 to PIP2, leading to cell apoptosis (24). It has been demonstrated that deficiency of PTEN resulted in activation of Akt, followed by phosphorylation of its downstream substrates (25). HDM2 is an important substrate of Akt, which functions as a negative regulator of tumor suppressor p53. Akt promotes nuclear location of HDM2, which is crucial for HDM2 to inhibit transcriptional activity of p53 and target p53 for degradation (26). Thus, PTEN may exert inhibitory effects on p53 through regulating Akt signaling, which promotes the translocation of HDM2 (27).

PTEN is dysregulated in CCRCC (8,17) and a deficiency of PTEN was correlated with poor prognosis in patients with advanced CCRCC (28). All these data indicated that loss of PTEN is important during CCRCC development, while it has remained elusive whether it is involved in chemoresistance of CCRCC. The present study assessed the expression of PTEN in CCRCC. Furthermore, the effects of short hairpin (sh)RNA-mediated PTEN knockdown in ACHN cells on Akt/HDM2 signaling, apoptosis induced by etoposide, cell proliferation, the interaction between HDM2 and p53, and the expression of p53 were evaluated. The present study illustrated that low expression of PTEN in CCRCC contributes to chemoresistance through activation of the Akt/HDM2 signaling pathway.

## Materials and methods

### Cell lines and cell culture

ACHN cells were purchased from the Cell Bank of Chinese Academy of Sciences (Shanghai, China) and cultured in RPMI 1640 supplemented with 10% fetal bovine serum (FBS), 100 U/ml penicillin and 100 U/ml streptomycin. Cells were maintained in a humidified incubator at 5% CO_2_ at 37°C (29).

### Human CCRCC and paired normal renal tissues

Five primary CCRCC and paired normal renal tissues were collected, between January 2010 and July 2011, from the Second Hospital, Jilin University (Changchun, China; 4 male and 1 female). All cases were confirmed clinical and pathologically and staged in accordance with the 2009 TNM staging classification system (30). The samples were snap-frozen in liquid nitrogen and stored at −80°C until further analysis or fixed in 4% paraformaldehyde. Written informed consent was obtained from all the patients or their guardians, and the protocol was approved by the Ethics Committee of the Second Hospital affiliated to Jilin University (Changchun, China).

### Reagents and antibodies

Antibody against phosphorylated (p)-HDM2 (Ser166; 1:1,000, Rabbit polyclonal antibody, cat. no. 3521) was purchased from Cell Signaling Technology (Danvers, MA, USA). Antibodies against total (T)-Akt (1:1,000, Rabbit polyclonal; cat. no. ab8806), p-AKT (Ser473; 1:1000, Rabbit Polyclonal; cat. no. ab66138), HDM2 (1:1,000, Mouse monoclonal; cat. no. ab10567), PTEN (1:1,000, Rabbit polyclonal, cat. no. ab31392) and p53 (1:1,000, Rabbit monoclonal; cat. no. ab179477) were purchased from Abcam (Cambridge, UK); poly (ADP ribose) polymerase (PARP cleaved), p53 upregulated modulator of apoptosis (PUMA, 1:1,000; mouse monoclonal; cat. no. sc-374223), GAPDH (1:1,000; mouse monoclonal; cat. no. sc-365062), rabbit anti-mouse immunoglobulin (Ig) G-horseradish peroxidase (HRP; 1:2,000; cat. no. sc-358922) and mouse anti-rabbit IgG-HRP (cat. no. sc-2357) were obtained from Santa Cruz Biotechnology, Inc. (Dallas, TX, USA). Protein A/G-beads and the enhanced chemiluminescence (ECL) immunoblotting detection reagent were purchased from Santa Cruz Biotechnology, Inc.; Etoposide was from Sigma-Aldrich (St Louis, MO, USA); Lipofectamine 2000 and TRIzol were purchased from Invitrogen Life Technologies (Carlsbad, CA, USA); Moloney murine leukemia virus (M-MLV) reverse transcriptase was from Promega (Madison, WI, USA). SYBR polymerase chain reaction (PCR) premixture was from Applied Biosystems (Foster City, CA, USA). Cell Counting Kit-8 was from Dojindo Laboratories (Kumamoto, Japan). pSUPERIOR.puro plasmid was obtained from Oligoengine (Seattle, WA, USA).

### Plasmid construction and transfection

The PTEN (GenBank accession number, NM_000314; AAGACCATAACCCACCACAGC) shRNA target sequences were designed by GeneChem Company (Montreal, Quebec, Canada) (forward, 5′-GACCAUAACCCACCACAGCTT-3′ and reverse, 5′-GCUGUGGUGGGUUAUGGUCTT-3′). The oligonucleotide-annealed products were subcloned into pSUPERIOR.puro (Oligoengine) between the *Bgl*II and *Hind*III sites.

For transfection, ACHN cells were seeded onto a 96-well plate (5×10^3^ cells/well) and following 24 h, cells were transfected with the indicated shRNA using Lipofectamine 2000 according the manufacturer’s instructions.

### Quantitative (q)PCR analysis

The total mRNA of tissues and ACHN cells was prepared using TRIzol according to the manufacturer’s instructions. Reverse transcription was performed with M-MLV reverse transcription kit. qPCR assays were performed with iTaq Fast SYBR Green Supermix (Bio-Rad) on a 7500 Fast Real-Time PCR System (Applied Biosystems, Foster City, CA, USA). The primers for qPCR were as follows: PTEN 5′-GTTCAGTGGCGGAACTTGCAATCCT-3′ (forward) and 5′-TCCCGTCGTGTGGGTCCTGA-3′ (reverse). Cycling conditions were as follows: 40 cycles of 95°C for 15 sec; 95°C for 20 sec and 60°C for 1 min. ^ΔΔ^CT method was used for the quantification of the products.

### Western blot analysis

Tissues or cells were collected and homogenized in radioimmunoprecipitation (RIPA) lysis buffer [50 mM Tris-HCl (PH 7.4), 150 mM NaCl, 1 mM phenyl methyl sulphonyl fluoride (PMSF; Beyotime Institute of Biotechnology, Shanghai, China), 1% Triton X-100 (Beyotime Institute of Biotechnology), 0.1% SDS (Amresco, OH, USA), 1% sodium deoxycholic acid (Amresco) and 1X protease inhibitor cocktail (Sigma-Aldrich)]. Then the homogenate was centrifuged at 7,300 × g for 10 min and the supernatant was collected. The protein concentration was measured using the bicinchoninic acid (BCA) protein assay kit (Beyotime, Shanghai, China). 40 *µ*g protein was subjected to 12% SDS-PAGE (Beyotime Institute of Biotechnology) and then electrophoretically transferred to a polyvinylidene difluoride (PVDF) membrane (Millipore Corp., Billerica, MA, USA). Following blocking with 5% skimmed milk (Amresco) for 1 h at room temperature, the membrane was incubated with the primary antibody for 1 h at room temperature. The membrane was washed with Tris-buffered saline containing Tween 20 (TBST; Beyotime Institue of Biotechnology) three times for 5 min each time. Then the membrane was incubated with HRP-conjugated secondary antibody for 1 h at room temperature. Following three washes with TBST, the positive signal was visualized using ECL Advanced Solution (Pierce, Thermo Fisher Scientific, Waltham, MA, USA).

### Immunoprecipitation

A total of 10 *µ*l protein A-sepharose slurry (Santa Cruz Biotechnology, Inc.) was added to the lysate prepared as above. The mixture was agitated at 4°C for 30 min and then centrifuged at 7,200 × g for 5 min. The supernatant was collected and transferred to a fresh Eppendorf tube. Subsequently, 2 *µ*g of the indicated antibodies and 20 *µ*l of the protein A-sepharose slurry were added to the pre-cleared lysate, and incubation was performed overnight at 4°C with continuous agitation. The immunocomplexes were washed three times with pre-cooled RIPA lysis buffer and precipitated proteins were eluted by boiling in 20–40 *µ*l 1X SDS-PAGE loading buffer (Beyotime Institute of Biotechnology). The samples were then subjected to western blot analysis.

### Immunohistochemistry

Tissues were fixed with parafor-maldehyde (4%; Sigma-Aldrich), then embedded in paraffin and sectioned. Sections were deparaffinized in xylol (Beyotime Institute of Biotechnology) and rehydrated in a graded ethanol series (China National Medicines, Shanghai, China). Following antigen retrieval by microwave heating (600 W; 15 min; P70F23P-G5; Galanz, Guangdong, China), the sections were incubated in non-immune serum (Santa Cruz Biotechnology, Inc.) for 30–60 min at room temperature and then blotted with the indicated primary antibody against p-Akt, p-HDM2 (Ser166), p53 or PTEN, overnight at 4°C. The next day, the sections were incubated in rabbit HRP-conjugated secondary antibody for 1 h at room temperature.

Finally, the sections were visualized with 3,3′-diamino-benzidine hydrochloride (Sigma-Aldrich) and counterstained with hematoxylin (Beyotime Institute of Biotechnology).

### Cell cycle analysis

Cells were detached with 0.25% trypsin (Beyotime Institute of Biotechnology), collected, washed with ice-cold phosphate-buffered saline (PBS; Beyotime Institute of Biotechnology) and then fixed in 70% ice-cold ethanol for 1 h. The cell suspension was centrifuged for 5 min at 1,000 rpm. The supernatant was removed and cells were re-suspended in PBS. Subsequently, 50 *µ*g/ml RNase A (Promega) and 25 *µ*g/ml propidium iodide (PI; Sigma-Aldrich) were added and kept at 37°C for 30 min. The DNA contents of >10,000 cells were detected using a FACSCalibur (BD Biosciences, San Jose, CA, USA). Quantitative analysis of the cell cycle distribution was performed using WinMDI 2.9 (Dr Joseph Trotter, The Scripps Institute, La Jolla, CA, USA).

### CCK8 assay

24 h prior to transfection, 2.5×10^3^ cells were seeded onto 96-well cell culture plates. Transfection was performed using Lipofectamine 2000 according to the manufacturer’s instructions. 24 h post-transfection, 30 *µ*M etoposide or mock were added. Following a further 24 h incubation, 10 *µ*l CCK-8 solution was added to each well of the plate, followed by 1 h incubation at 37°C. The optical density (OD) was then measured at 450 nm using a microplate reader (MK3; Thermo Fisher Scientific). The cell inhibitory rate was calculated according to the following equation: [1−(OD_experiment_−OD_blank_)/(OD_contro_l−OD_blank_)] ×100%. All experiments were performed in triplicate and repeated three independent times.

### Statistical analysis

Values are expressed as the mean + standard error. Student’s t-test was used for statistical analysis of the data to determine the differences between the groups using SPSS 11.5 for Windows software (SPSS, Inc., Chicago, IL, USA). P<0.05 was considered to indicate a statistically significant difference between values.

## Results

### PTEN is overexpressed in CCRCC tissues

**Figure 1:**
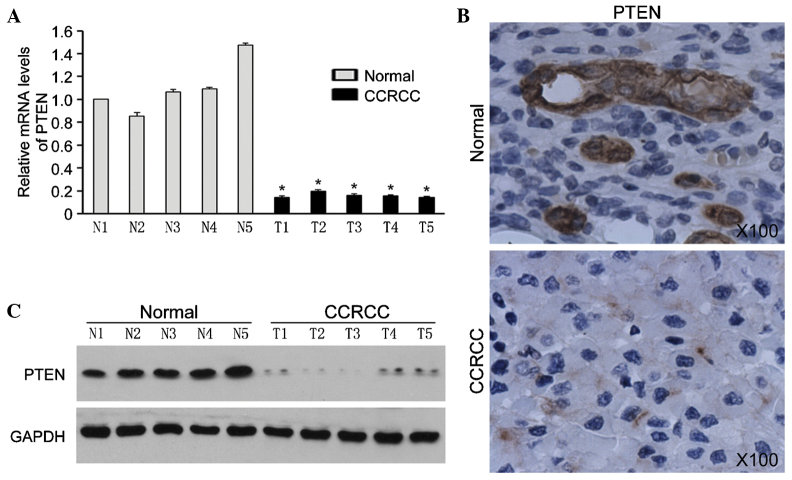
Expression of PTEN in CCRCC and non-tumor kidney tissues (N1–N5). (A) Expression of PTEN mRNA was detected using reverse transcription quantitative polymerase chain reaction. mRNA levels were normalized to GAPDH expression. Values are expressed as the mean + standard deviation of three independent experiments performed in triplicate. T1–T5, CCRCC samples; N1–N5, normal renal tissue samples ^*^P<0.001 compared with normal tissues. (B) Immunohistochemical analysis for PTEN protein expression in CCRCC and non-tumor kidney tissues. Representative micrographs are shown (magnification, x100). (C) Protein expression of PTEN in CCRCC and non-tumor kidney tissues by western blot analysis. GAPDH was used as a loading control. PTEN, phosphatase and tensin homolog; CCRCC, clear-cell renal cell carcinoma.

In the present study, the expression of PTEN was analyzed in five pairs of CCRCC tissues and the corresponding normal renal tissues. qPCR analysis showed that PTEN mRNA was decreased in CCRCC tissues (Fig. 1A). In accordance with the qPCR results, western blot analysis and immunohistochemistry showed that the expression levels of PTEN protein were obviously decreased in CCRCC tissues (Fig. 1B and C). These results suggested that loss of PTEN is correlated with CCRCC development.

### PTEN knockdown inhibits ACHN cell apoptosis induced by etoposide

**Figure 2:**
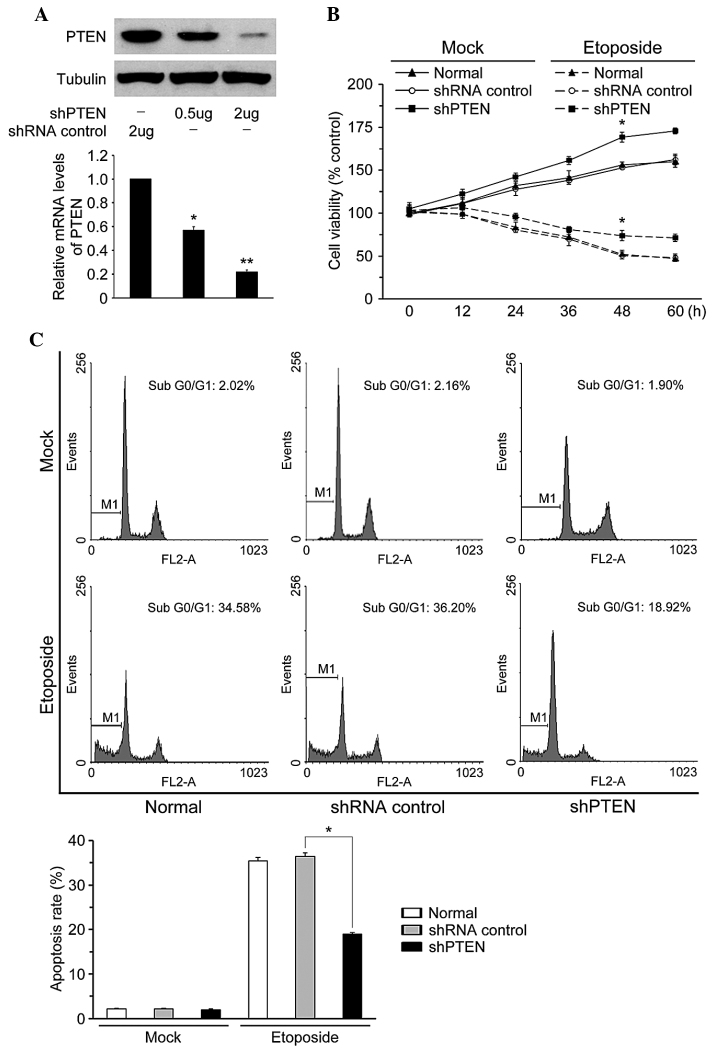
Effects of knockdown of PTEN on etoposide-induced DNA damage response and cell growth inhibition. (A) Expression levels of PTEN after stable shRNA silencing in ACHN cells. (B) PTEN knockdown increased the viability of ACHN cells upon treatment with or without 30 *µ*M etoposide for 24 h. (C) PTEN silencing inhibited etoposide-induced cell cycle arrest and apoptosis in ACHN cells. The bar graph shows apoptotic rates of cells following PTEN silencing in the presence or absence of etoposide. Corresponding cell cycle phase distributions are shown in the top panel. (^*^P<0.05, ^**^P<0.01) shRNA, small hairpin RNA; PTEN, phosphatase and tensin homolog.

**Figure 3:**
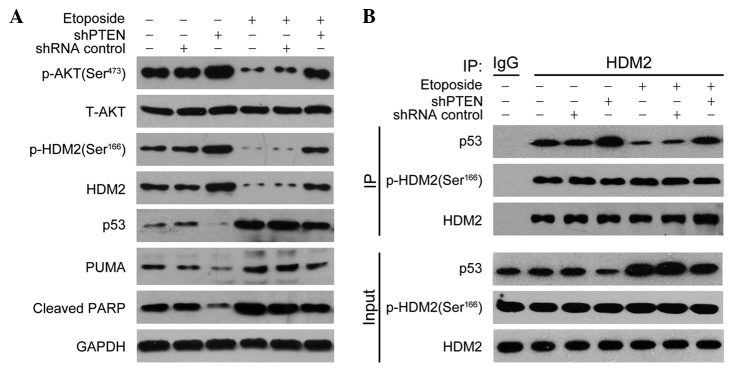
Effect of etoposide on the expression of different endogenous proteins of the Akt pathway in ACHN cells. (A) Western blot analysis of cleaved-PARP, PUMA, p-AKT (Ser473), T-AKT, p-HDM2, HDM2 and p53 in ACHN cells with indicated treatment. GAPDH was loaded as the internal control. (B) Co-immunoprecipitation of HDM2 in normal cells or clear-cell renal carcinoma cells were precipitated for HDM2 and immunoblotted for p53, p-HDM2 and HDM2 in ACHN cells with indicated treatment. shRNA, small hairpin RNA; PTEN, phosphatase and tensin homolog; p, phosphorylated; T, total; PUMA, p53 upregulated modulator of apoptosis; IgG, immunoglobulin G; PARP, poly(ADP ribose) polymerase; I P, immunoprecipitation.

To identify whether PTEN has a role in the sensitivity of CCRCC to chemotherapy drugs, PTEN was silenced in the CCRCC cell line ACHN using shRNA. As shown in Fig. 2A, following transfection with PTEN shRNA, PTEN was effectively downregulated in ACHN cells. Results of the CCK-8 assay showed that knockdown of PTEN promoted proliferation of ACHN cells with or without treatment with etoposide (Fig. 2B). Cell cycle analysis revealed that treatment with etoposide induced cell cycle arrest and apoptosis in ACHN cells, while PTEN knockdown resulted in a decreased sub G0/G1 phase population in the presence or absence of etoposide (Fig. 3C). These results demonstrated that loss of PTEN in ACHN cells inhibited etoposide-induced cell apoptosis and promoted cell proliferation.

### PTEN inhibition leads to activation of AKT/HDM2 and downregulation of p53

PTEN is a specific antagonist of PIP3, which blocks PI3K/Akt signaling through dephosphorylation of PIP3 (24). Increasing evidence showed that loss of PTEN resulted in abnormal activity of Akt (31). In the present study, etoposide-treatment of ACHN cells increased phosphorylation of Akt (Fig. 3A); furthermore, HDM2, an important substrate of Akt, was significantly inhibited. p53, PUMA and cleaved PARP were obviously induced. Knockdown of PTEN in ACHN greatly reversed the inhibition effect of etoposide on Akt and HDM2 activation. The etoposide-induced upregulation of p53, PUMA and cleaved PARP were also reduced following PTEN knockdown. These results suggested that PTEN knockdown activated the Akt/HDM2 signaling pathway and blocked p53-dependent cell apoptosis (Fig. 3A).

HDM2 is a negative regulator of tumor suppressor p53, which inhibits the transcriptional activity of p53 and targets p53 for degradation (26). Immunoprecipitation results showed that knockdown of PTEN enhanced the interaction between p53 and HDM2 (Fig. 3B). The results further demonstrate that loss of PTEN in ACHN cells activated the Akt/HDM2 signaling pathway and promoted the interaction between p53 and HDM2, which led to degradation of p53 and resulted in block of apoptosis induced by etoposide.

### Activation of AKT/HDM2 leads to degration of p53 and enhances cell proliferation

**Figure 4:**
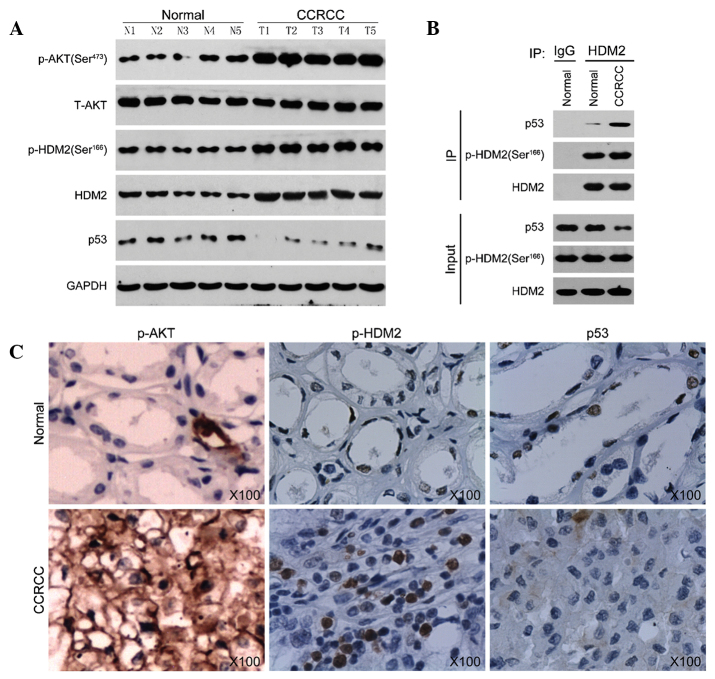
HDM2 interacts with p53 in CCRCC. (A) Protein levels of p-AKT (Ser473), T-AKT, p-HDM2, HDM2 and p53 in non-tumor kidney tissues (normal, N1–N5) and CCRCC (T1–T5). GAPDH was used as the internal control. (B) Co-immunoprecipitation of HDM2 in normal cells or CRCC were precipitated for HDM2 and immunoblotted for p53, p-HDM2 and HDM2. (C) Representative micrographs of immunohistochemical staining for p-AKT (Ser473), p-HDM2 and p53 in non-tumor kidney tissues and CCRCC (magnification, x100). CCRCC, clear-cell renal cell carcinoma; p, phosphorylated; T, total; IgG, immunoglobulin G; I P, immunoprecipitation.

To further verify that PTEN deficiency is critical in chemoresistance of CCRCC, the Akt/HDM2 signaling pathway in CCRCC tissues was analyzed. Western blot analysis showed that HDM2, p-HDM2 (ser166) and p-AKT were upregulated in CCRCC tissues alongside low p53 expression (Fig. 4A). The immunohistochemistry results were in accordance with the western blot results (Fig. 4B). Furthermore, immunoprecipitation also revealed that interaction between p53 and HDM2 was increased in CCRCC tissues. These data confirmed that loss of PTEN in CCRCC is attributed to activation of Akt/HDM2 and enhancement of the interaction between p53 and HDM2, eventually resulting in a reduction of p53.

## Discussion

In the present study, it was found that PTEN was depressed in CCRCC, which was involved in the resistance of CCRCC to chemotherapy. Further investigation revealed that loss of PTEN activated Akt/HDM2 and promoted degradation of p53, which attributes to resistance of CCRCC cell apoptosis induced by etoposide.

PTEN is a dual and protein phosphatase, and it has been demonstrated that loss of PTEN is closely associated with tumorigenesis. In the present study, it was also found that the expression of PTEN protein and mRNA was downregulated in CCRCC, which was in accordance with the results of previous studies (8,17). The present study suggested that loss of PTEN has an important role during CCRCC development. Etoposide is a commonly used anti-tumor drug, which induces apoptosis of numerous types of tumor cell. Treatment of ACHN with etoposide enhanced cell apoptosis and inhibited cell proliferation. Knockdown of PTEN in ACHN cells using PTEN shRNA increased cell proliferation, and the apoptotic rate decreased from 34.58 to 18.92%. These results demonstrated that deficiency of PTEN is associated with chemoresistance of CCRCC.

Tumor suppressor gene p53 has an important role in regulating cell cycle and cell apoptosis and is frequently mutated or deleted in various tumors. The mechanism of action of most chemotherapy drugs is the induction of p53-mediated apoptosis. Therefore, tumors harboring p53 mutations are associated with chemoresistance and poor prognosis (32–34). While p53 mutations are seldom detected in CCRCC, these tumors are frequently resistant to chemotherapy (35–37). The present study found that p53 and PUMA were overexpressed in etoposide-treated ACHN cells, and the percentage of apoptotic cells significantly increased, which suggested that etoposide induced apoptosis of ACHN cells in a p53-dependent manner.

The PI3K/Akt signaling pathway has a key role in the development of numerous tumor types, including CCRCC (17). PTEN is an antagonist of PI3K, which dephosphorylates PIP3 and blocks signaling downstream of activated PI3K (24). HDM2 is a substrate of PI3K/Akt and also a ubiquitin ligase of p53. Akt phosphorylates serines 166 and 186 on HDM2, which induces translocation of HDM2 from the cytoplasm into the nucleus where it binds to p53 and promotes p53 degradation (38–40). To elucidate the mechanism underlying the role of PTEN deficiency in CCRCC resistance to chemotherapy, the present study examined the PI3K/Akt signaling pathway in etoposide-treated ACHN cells. The results showed that etoposide was able to inhibit the activation of Akt and HDM2, and the expression of p53 and PUMA was also depressed. Furthermore, an immunoprecipitation assay showed that the interaction between HDM2 and p53 was reduced. From the above data it was deduced that in CCRCC, loss of PTEN may activate the Akt/HDM2 interaction, leading to suppression of p53 and hence blocking apoptosis induced by chemotherapy. This was confirmed by knockdown of PTEN in ACHN cells, as decreased expression of PTEN in ACHN cells markedly reversed the etoposide-mediated inhibition of Akt/HDM2 and led to downregulation of p53.

Finally and importantly, the present study examined Akt/HDM2 signaling in CCRCC tissues and also found that HDM2, p-HDM2 (ser166) and p-Akt were increased, accompanied with depressed p53. Furthermore, the amount of p53 co-precipitated with HDM2 in CCRCC was obviously increased. These results further demonstrated that CCRCC resistance to chemotherapy caused by PTEN deficiency was associated with the Akt/HDM2 signaling pathway.

In conclusion, the present study illustrated that loss of PTEN in CCRCC led to activation of the Akt/HMD2 interaction, inhibition of p53 and protection of CCRCC cells from etoposide-induced apoptosis. These results provided a mechanistic explanation of why CCRCC cells with PTEN deficiency are resistant to chemotherapy and support a rationale for combining conventional chemotherapy drugs, such as etoposide, with modalities that activate p53 for the efficient treatment of CCRCC.
